# Postcardiac arrest syndrome: from immediate resuscitation to long-term outcome

**DOI:** 10.1186/2110-5820-1-45

**Published:** 2011-11-03

**Authors:** Nicolas Mongardon, Florence Dumas, Sylvie Ricome, David Grimaldi, Tarik Hissem, Frédéric Pène, Alain Cariou

**Affiliations:** 1 Medical Intensive Care Unit, Cochin Hospital, Groupe Hospitalier Universtitaire Cochin Broca Hôtel-Dieu, Assistance Publique des Hôpitaux de Paris, 27 rue du Faubourg Saint-Jacques, 75014 Paris, France; 2 Université Paris Descartes, Sorbonne Paris Cité, Faculté de Médecine, 15 rue de l'Ecole de Médecine, 75006 Paris, France; 3 INSERM U970, Cardiovascular Research Center, European Georges Pompidou Hospital, 56 rue Leblanc, 75015 Paris, France

## Abstract

The prognosis for postcardiac arrest patients remains very bleak, not only because of anoxic-ischemic neurological damage, but also because of the "postcardiac arrest syndrome," a phenomenon often severe enough to cause death before any neurological evaluation. This syndrome includes all clinical and biological manifestations related to the phenomenon of global ischemia-reperfusion triggered by cardiac arrest and return of spontaneous circulation. The main component of the postcardiac arrest syndrome is an early but severe cardiocirculatory dysfunction that may lead to multiple organ failure and death.

Cardiovascular support relies on conventional medical and mechanical treatment of circulatory failure. Hemodynamic stabilization is a major objective to limit secondary brain insult. When the cause of cardiac arrest is related to myocardial infarction, percutaneous coronary revascularization is associated with improved prognosis; early angiographic exploration should then be discussed when there is no obvious extracardiac cause. Therapeutic hypothermia is now the cornerstone of postanoxic cerebral protection. Its widespread use is clearly recommended, with a favorable risk-benefit ratio in selected population. Neuroprotection also is based on the prevention of secondary cerebral damages, pending the results of ongoing therapeutic evaluations regarding the potential efficiency of new therapeutic drugs.

## Introduction

Sudden death remains a major public health issue, despite improvements in prehospital management and standardization of advanced life support through wide diffusion of international guidelines [1]. Both incidence and poor prognosis are striking: according to official statistics, approximately 100,000 people are supported for out-of-hospital cardiac arrest (OHCA) in the United States each year. However, it is estimated that the real number of sudden death is two to three times higher. Even more problematic, less than 10% of patients admitted to the hospital after successfully resuscitated OHCA will leave the hospital without major neurological impairments.

For patients who survive the initial phase of prehospital care, the course is usually marked by two types of events:

1. Syndrome originally described as an early reperfusion syndrome (or "postresuscitation disease"), which usually appears between the 4th and 24th hour in the form of a stereotypical feature whose extreme form involves a state of shock, high fever, and severe biological disorders [2].

2. Poor neurological prognosis because two thirds of patients who survive the early phase will subsequently develop neurofunctional sequellae, which sometimes progress toward a postanoxic vegetative state and delayed death [3].

The frequency and intensity of these complications depend largely on the delay of initial treatment, the efficiency of resuscitation, and the time elapsed between collapse and return of spontaneous circulation (ROSC). At that time, CA and its resuscitation is the clinical situation closest to the phenomenon of "ischemia-reperfusion," well known from experimental models [4]. Moreover, this is the only medical situation that enables measurement of clinical acute consequences of global ischemia, targeting simultaneously all tissues and organs. The pathophysiology of this syndrome explains the observed features and justifies the therapeutic interventions that must be performed to achieve a favorable neurological evolution. Optimizing postresuscitation care is of paramount importance, because it represents the last link of the survival chain.

## Pathophysiology of postcardiac arrest syndrome

**Figure 1:**
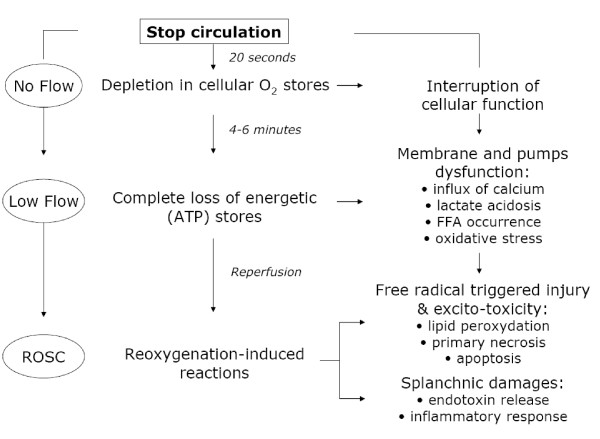
**Schematic pathophysiology of postresuscitation damages**.

The pathophysiology of postcardiac arrest syndrome is complex and remains partially understood (Figure 1). It seems, however, dominated by a global ischemia-reperfusion phenomenon (affecting all organs) and a nonspecific activation of the systemic inflammatory response. During ischemia (phase of "no flow"), reduced oxygen supply is offset by lower metabolic needs. However, if cell metabolism remains requested or if the ischemia period is prolonged, the decrease of ATP synthesis leads to a plasma membrane depolarization, opening of voltage-dependent calcium channels sarcolemne, and fall of mitochondrial membrane potential. Schematically these phenomena result in an increase of intracytoplasmic calcium concentration responsible for cellular damage. Thus, it is during the phase of "no flow" that the first cell and tissue damage will occur. Reperfusion (phase of "low flow"), contemporary to the restoration of blood flow (created by chest compression, or spontaneous), is responsible for the formation of oxygen radical species, including superoxide anion (• O2-), hydrogen peroxide (H_2_O_2_) and the radical hydroxyl (• OH). The latter, particularly cytotoxic, carries for most functional and structural lesions causing cell death [4]. Indeed, it inactivates cytochromes, alters membrane transport proteins, and induces phenomena of membrane lipid peroxidation. *In vivo *demonstration was provided by studying plasma of out-of-hospital CA survivors, which induced acute and major endothelial toxicity, due to an acute pro-oxidant state that occurs within the cells, and illustrated by a decrease in antioxidant activity [5]. Disseminated vascular endothelium damages explain that this phenomenon of ischemia-reperfusion evolves toward systemic inflammation: production of cytokines (IL-1, IL-6, IL-8, TNF-α), complement activation, synthesis of metabolites of arachidonic acid, expression of leukocyte adhesion molecules by endothelial cells are all stimuli for activation, and chemotaxis of polymorphonuclear neutrophils at the origin of the inflammatory response. Sequestration of activated neutrophils in the lungs and other organs is a major engine for development of multiple organ failure. It is plausible that infectious lesions of digestive origin are involved, in relation to bacterial translocation from altered gastrointestinal mucosa. It would explain the high levels of plasma endotoxin observed in the most critically ill patients. This activation of the systemic inflammatory response associates with changes in coagulation, generating secondary endothelial damage, in turn responsible for thrombosis and increased capillary permeability [6]. Experimental models of CA support the pathophysiological hypothesis of worsening of visceral lesions that occur during the reperfusion phase and extend over the first hours, explaining the efficiency of some delayed therapeutic interventions (as therapeutic hypothermia). In humans, there is an activation of the inflammatory reaction associated with clinical features that are very similar to those observed during severe sepsis [7]. Coagulation abnormalities have been identified, involving a significant activation of coagulation factors, whereas endogenous anticoagulants (antithrombin, protein S and C) are decreased [8]. This intravascular coagulation can be implicated in the genesis of microvascular abnormalities, which in turn lead to more visceral lesions. Of note these coagulation abnormalities are particularly common in patients who die quickly from a post-resuscitation shock.

## Clinical features

The postcardiac arrest syndrome presents a set of relatively stereotyped events [9]. Its intensity varies but is roughly proportional to the duration of "no flow" and "low flow," as well as difficulty encountered during initial resuscitation [10]. The cardiocirculatory failure usually dominates the clinical picture, even if it often leads to multiorgan failure (MOF). The decrease of cerebral perfusion, caused by these hemodynamic disturbances, might further impair the neurological prognosis of these patients.

## Postcardiac arrest shock

The postcardiac arrest shock, initially described by Negovsky in 1975, is a mixed shock, with cardiogenic and vasodilatory components [2] and is characterized by a severe but reversible systolic dysfunction. Left ventricular dysfunction usually begins early, within minutes after ROSC, and is completely reversible within 48 to 72 hours. It takes the form of myocardial systolic and diastolic dysfunctions, even in the absence of coronary cause. The experimental evidence of this early, intense, and transient myocardial dysfunction after CA could be confirmed in humans. Indeed, Laurent et al. described the hemodynamic profile of postcardiac arrest shock [11]. In this study including 148 consecutive patients after a successfully resuscitated CA, 73 patients (49%) developed an early shock (within a median time of 8 hours after initial resuscitation) and were explored invasively with pulmonary artery catheter. Upon admission to the ICU, these patients exhibited a significant decrease in angiographic left ventricular ejection fraction, regardless of the etiology of CA. This shock was characterized by a low cardiac output and normal or low left filling pressures. Interestingly, in agreement with experimental data, the cardiac index improved quickly in patients with shock, from 2.1 l/min/m^2 ^(median value at the 8^th^ hour) to 3.2 l/min/m^2 ^at the 24^th ^hour, and up to 3.7 l/min/m^2 ^at the 72^th ^hour. The persistence of a low cardiac index was associated with early death, usually in a setting of MOF. But the circulatory failure does not appear to be related only to myocardial dysfunction. Indeed, despite the fast improvement of contractile function in survivors, this study also showed that vasopressors often had to be maintained until the 72^th ^hour, in association with important fluid infusion to maintain adequate filling pressures. Thus, these data support the existence of an early and intense myocardial failure, usually regressive within 48 hours, secondarily associated with a severe vasodilatation, as a result of the generalized inflammatory syndrome, itself well documented after CA [6]. Obviously, this has to be distinguished from the focal hypocontractility caused by a coronary thrombosis, which evolves on its own. The circulatory failure might also be favored by the occurrence of relative adrenal insufficiency, as observed during septic shock: clinical data show that approximately half of patients have relative adrenal insufficiency similar to what can be observed in septic shock. Among these patients, shock-related deaths seem to be more frequent in the group of those with adrenal insufficiency [12].

## Neurological failure

The anoxic-ischemic neurological damages remain the leading cause of deaths occurring in patients initially resuscitated from CA. This neurological impairment can usually be detected from the third day after initial resuscitation. However, the dogma that the brain damage would be only due to injuries occurring contemporarily to circulatory interruption has been widely challenged during the past decade. Clinical studies that confirmed the efficiency of therapeutic hypothermia despite its "late" initiation, as regard to the onset of CA, support the existence of a worsening process during the reperfusion period. Indeed, neurological damage is initiated during circulatory interruption but also is accentuated during the reperfusion phase. Experimental and clinical studies show that the cerebral blood flow is decreased during the postcardiac arrest syndrome, associated with a decrease in cerebral oxygen extraction. The reduction of cerebral blood flow, which seems to be related to a transient increase in cerebral vascular resistance, will be corrected within 72 hours. Among survivors, the normalization of cerebral blood flow is accompanied by an increase in cerebral oxygen extraction, reflecting the recovery of the physiological coupling of these two parameters [13]. However, in patients with lethal brain injuries, the difference in coupling between cerebral blood flow and extraction appears gradually and finally results in a "luxury" perfusion.

## Other organ failures

Without specific and prompt treatment, postcardiac arrest shock usually leads to a multiple organ failure and early death. Acute renal and respiratory dysfunctions occur in approximately 40-50% of patients resuscitated from CA. Hypoxemia (a consequence of pulmonary edema, but also lung contusion, atelectasia, or aspiration), cardiogenic shock, acute renal failure, and liver failure may of course worsen the prognosis and delay neurological recovery. The digestive tract insult often is underestimated, due to the lack of bedside tool to investigate its function; however, its alteration should not be considered, both as a victim of the circulatory failure and as a second source of organ failure through translocation and major source of endotoxemia.

## Infectious issues

Many mechanisms that occur after CA make cardiopulmonary resuscitation survivors prone to infectious complications. Loss of airways' protection, coma, pulmonary contusion, emergency airway and vascular access, mechanical ventilation, and ischemia-reperfusion increase the risk for infection. The diagnosis is extremely complex, due to many clinical, biological, and radiological confounding factors. Moreover, therapeutic hypothermia plays a dual role by promoting infection and by impairing both host defences and usual infectious criteria. Recently developed biomarkers, such as procalcitonin, perform poorly in the assessment of septic complications [14]. We recently demonstrated that two thirds of CA survivors presented infectious complications during their ICU stay and may be favored by hypothermia. Despite increase in mechanical ventilation duration and length of stay, mortality and neurological outcome were not impaired by infectious events [15]. If antibioprophylaxis is not recommended, infectious complications should be cautiously tracked, especially when therapeutic hypothermia is employed.

## Therapeutic aspects

**Figure 2:**
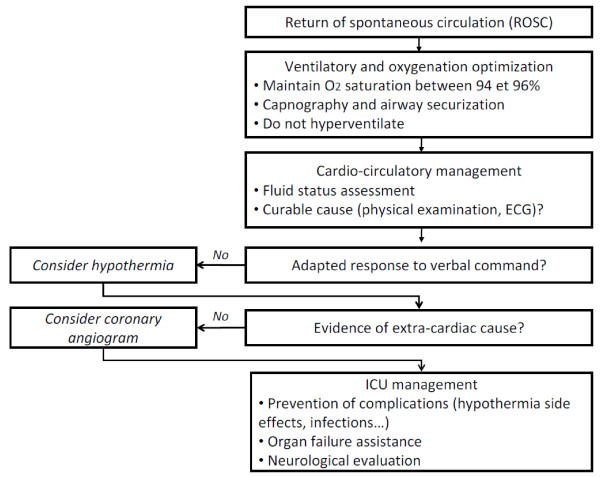
**Early postcardiac arrest management**.

The therapeutic management of the postcardiac arrest syndrome has two main goals: the initial treatment of shock and organ failures, and the optimization of cerebral protection. These two aspects are obviously very intricate, initial treatment having the essential interest to allow bringing the majority of patients to neurological evaluation, under hemodynamic stability conditions. The medical algorithm that we apply in our unit is displayed in Figure 2.

## Cardiovascular management

### Shock treatment

Except in special cases, the impossibility of early reliable neurological assessment justifies the introduction of organ support during the initial postresuscitation phase. When initially controlled, the postresuscitation shock is usually reversible within 48 to 72 hours and treatments differ little from inflammatory shock associated with heart failure. Regarding myocardial dysfunction, the treatments are the usual drugs of acute heart failure. The favorable effect of dobutamine on myocardial dysfunction after CA was confirmed by experimental models, demonstrating improved systolic and diastolic functions [16]. However, the restoration of ventricular contractility is accompanied by a dose-dependent tachycardia, which may cause a rise in myocardial oxygen consumption. The use of epinephrine often is necessary in the most severe forms of shock after cardiac arrest, because ventricular dysfunction is usually associated with severe vasoplegia at this phase. However, in addition to its usual side effects, epinephrine exhibits an increase in myocardial oxygen consumption and a potentially deleterious activation of metabolic intracellular signals. Association of dobutamine and norepinephrine could be preferred. In this situation, the correction of absolute or relative hypovolemia is crucial and should, whenever possible, precede the introduction of vasoconstrictors. Other inotropic drugs have been tested (phosphodiesterase inhibitors, levosimendan) but without demonstrating superior efficiency. Widely used for ischemic cardiogenic shock, intra-aortic counterpulsation reduces cardiac work by reducing left ventricular afterload and improves tissue perfusion and diastolic coronary perfusion. During postcardiac arrest shock, its interest is not clearly established [17]. The use of circulatory support by extracorporeal membrane oxygenation after cardiac arrest is experiencing growth and shows promising results on mortality [18,19]. However, its indications appear limited because of the cumbersome logistics of such therapy requiring a specialized team, in the setting of an unknown neurological outcome. Alternative techniques for minimally invasive circulatory support, such as the miniaturized system Impella^® ^(Cardiosystems Impella AG, Aachen, Germany) that uses a micropump assistance of the left ventricular introduced via the femoral artery, should be evaluated in this context.

### Correction of hemodynamic disorders

The particularities of postcardiac arrest shock suggest a clinical feature close to septic shock, suggesting a beneficial effect of extracorporeal treatment referred as "anti-inflammatory." A pilot study showed that renal replacement therapy by high-volume hemofiltration (with or without hypothermia) could help to reduce early mortality related to hemodynamic failure after cardiac arrest [20]. This improvement could be related to better control of cardiovascular failure in some patients. However, these preliminary data do not allow recommending the routine use of this technique in postcardiac arrest syndrome, and further studies are required in this way. Regarding the level of arterial pressure that should be targeted during this period, the single study conducted on this point failed to show significant difference between patients maintained at a mean arterial pressure > 100 mmHg vs. < 100 mmHg in the first 2 hours after ROSC [21]. Hemodynamic monitoring using at least arterial catheter seems essential; an echocardiography performed within the first 24 hours seems useful, not only for the etiological diagnosis but also to optimize the hemodynamic status of these patients. However, no monitoring device has definitively proven its superiority in this setting.

### Coronary revascularization

Acute coronary syndrome is the most common cause of out-of-hospital cardiac arrest in adults. A systematic application of coronary angiography on hospital arrival for all survivors of CA found a recent coronary occlusion in 49% of cases [22]. Clinical data (cardiovascular risk factors chest pain) and ECG perform poorly to predict coronary occlusion; in a recent study, 96% of patients with ST-segment elevation on the ECG performed after ROSC, and 58% of patients without ST-segment elevation had at least one significant coronary artery lesion [23]. Moreover, there was an independent association between successful dilatation of a coronary artery responsible for CA and hospital survival. Consequently, the indication of coronary angiography should be considered widely, as well as the prehospital referring of the patient to a center able to provide coronary angiography if required: current guidelines recommend performing immediate coronary revascularization in patients with OHCA [24]. The results obtained in routine clinical practice on research and systematic treatment of acute coronary occlusion in survivors of cardiac arrest in ischemic context also are particularly encouraging [23].

## Neuroprotection

The existence of further cerebral damage during the reperfusion phase encouraged intense research on the benefit of treatments designed to limit the neurological consequences of the postcardiac arrest syndrome.

### Therapeutic hypothermia

Many experimental data show that mild hypothermia can exert neuroprotective effects through multiple mechanisms of action: decrease of cerebral metabolism, reduction of apoptosis and mitochondrial dysfunction, slowing of the cerebral excitatory cascade, decrease of local inflammatory response, reduction of free oxygen radicals production, and decrease of vascular and membrane permeability. The convergent experimental data were confirmed by two landmark clinical studies published in 2002 [25,26]. In both trials, the implementation of mild hypothermia achieved a survival rate without major sequel in approximately 40-50% of a highly "selected" population (OHCA with an initial rhythm of ventricular fibrillation in front of a bystander). Hypothermia was achieved in both cases by external cooling methods (blanket, ice packs), associated with routine use of neuromuscular blocking agents. At the targeted levels of hypothermia (32-34°C), theoretical adverse effects (arrhythmias, coagulopathy, infection) were very rare. A trend toward greater frequency of bleeding and infectious events was observed but was greatly overweighed by the neurological and survival benefit. The publication of these two studies was decisive and led to a rapid change in international recommendations on the management of patients surviving after CA. It is now strongly recommended to use moderate hypothermia routinely (32-34°C) for 12 to 24 hours in any comatose adult successfully resuscitated after OHCA caused by ventricular fibrillation/tachycardia [1]. In patients presenting with an initial nonshockable rhythm, the level of evidence is weaker, and some recent data suggest a lack of neurological benefit [27]. Considering that the risk-benefit ratio is sufficiently favorable, it is reasonable to discuss the indication for treatment on a case-by-case basis. Finally, no single cooling method has been shown to be superior in terms of clinical outcomes.

### Neuroprotective drugs

Despite numerous attempts, no drug has clearly demonstrated its ability in the early or late phase of resuscitation to reduce the consequences of cerebral anoxo-ischemia after CA, despite encouraging animal data and early administration after ROCS. Similarly, the different sedative agents have not proven their efficiency. Thus, apart from special situations (therapeutic hypothermia for instance), there is no argument for recommending the routine use of pharmacological agents in CA survivors. Currently, several molecules with cytoprotective effects during ischemia-reperfusion phenomena are investigated. The more advanced research are available for cyclosporine, which shows promising preliminary results through inhibition of the opening of the mitochondrial permeability-transition pores; erythropoietin is as well under consideration, with a strong experimental background of polyfactorial neuroprotection and encouraging clinical outcomes [28,29].

## Prevention of secondary cerebral damages

Achieving and maintaining a perfect homeostasis, particularly in terms of metabolism, are the major goals of post cardiac arrest management.

### Hematosis control

Hypoxemia should be avoided by increasing the oxygen fraction of inspired gas to maintain arterial oxygen saturation > 92% to maintain a sufficient oxygen transport to peripheral tissues. It is not necessary to set a target of "supraphysiological" PaO_2_. Moreover, hyperoxia is a matter of debate [26,30]. The level of PaCO_2_, which is completely controlled by the ventilatory settings in this sedated and sometimes paralyzed patient, is exposed to significant variations. Hypocapnia, meanwhile, should be avoided because it causes a reduction of cerebral blood flow. After animal CA, ROSC is accompanied by a transient cerebral hyperemia by approximately 15-30 minutes, followed by a secondary and more prolonged decline. In a canine model of CA using therapeutic hypothermia, hyperventilation appeared to worsen the neurological outcome [31]. In addition, hyperventilation may be responsible for increased intracranial pressure by increasing positive end-expiratory pressure. Conversely, hypercapnia, leading to cerebrovascular vasodilatation and increased intracranial pressure, should be banned. Overall, despite the absence of clinical studies that specifically address the ventilatory settings, it seems logical to maintain a PaCO_2 _within normal limits [9]. For this, it is necessary to monitor the quality of ventilation using, whenever possible, measurement of expiratory tidal CO_2 _and regular monitoring of arterial blood gases, especially during cooling and warming phases, which significantly alter production of CO_2_.

### Metabolic control

The correction of electrolyte disturbances is essential and special attention should be given to the one that may participate in the recurrence of CA or worsening of organ dysfunction. A potential survival benefit was recently demonstrated, when tight-controlled glycemia was applied to an overall population of patients admitted to surgical and medical intensive care. Co-administration of glucose and insulin has been demonstrated as beneficial on the neurological recovery in an animal model of CA [32]. Even if there is a strong association between high blood glucose levels and adverse neurological outcome after CA, conclusions on association or causality cannot be drawn. The increased frequency of hypoglycemia observed during tight control glycemia should be kept in mind before extending this therapeutic approach in patients with severe brain damage and possibly more sensitive to the deleterious consequences of a hypoglycemic episode. Thus, in the absence of additional data, it is not possible to recommend the use of tight control glycemia in patients with postcardiac arrest shock. Such an attitude should be demonstrated in terms of benefit/risk ratio. However, converging data underline that blood glucose variability more seriously impairs the outcome of critically ill patients rather than the mean level of glycemia. This observation has recently been confirmed in cardiac arrest survivors [33], so that attention should probably be paid to avoid such glycemia fluctuations.

## Outcome prediction

Immediately after CA, no clinical signs or investigations accurately predict the patient's outcome. During the first hours and days after ROSC, half of these patients will subsequently suffer from a postresuscitation disease, which can include a severe shock leading by itself to death. As mentioned earlier, shock should be treated without limitation during the very first hours and days to reach the adequate window for neurological evaluation. Finally, brain death occurs in approximately 10% of these patients a few days after CA because of cerebral edema [34]. Those patients are dead and life support will be stopped after completion of the organ-donation process.

After ROSC, accurate prognostication can only occur after 72 hours have elapsed from all factors that may alter neurological evaluation. This last point is of particular importance if sedation and neuromuscular blockade were used in conjunction with therapeutic hypothermia, because this treatment strategy must extend the waiting period [35]. Thus, if hypothermia is employed, it also is reasonable to use short-acting drugs. All other usual confounding situations (medications, hypothermia, and concomitant organ failure) must be resolved before any prognostication process.

## Tools to predict outcome during ICU stay

Accurate assessment of prognosis is necessary to identify patients who will really benefit from intensive care and to avoid extending unnecessary treatments to those who have no reasonable chance of recovery. Prognostication should always be based on a rigorous clinical evaluation, which may include detailed neurological examination. Because there is no specific clinical sign that can predict outcome in the first few hours after ROSC, reliable prognostic factors and assessment of the ICU admission could be very useful. At this very early stage, a few elements are however available, and their accuracy are sometimes questionable. These elements can be grouped roughly into four main categories: intrinsic patient characteristics (age and comorbidities), parameters reflecting the quality of prehospital care ("no flow" and "low flow" durations), the etiology of CA, and finally, some of the patient's clinical characteristics:

- Intrinsic characteristics, including age, have a modest prognostic weight on early survival [36];

- The speed and quality of prehospital care logically have a major impact on the prognosis of patients. If "no flow" and "low flow" durations are valuable prognostic factors, with substantial mortality over respectively 5 and 15 minutes [36], they cannot carry a final decision element. In addition, the accuracy of the information collected often is questionable in this context, which limits the scope of their prognosis ability.

- Concerning the cause of CA, asphyxia, and to a lesser degree, all extracardiac causes, are predictors of poor prognosis. In contrast, sudden cardiac death, occurring on a body properly oxygenated, is usually associated with better prognosis. However, once again, decisions cannot be made according to this oversimplistic split.

The absence of pupillary light response is not sufficiently predictive when it is sought on admission, due to a high false-positive rate, which can reach 30% [37]. Several additional clinical findings accurately predict poor outcome: myoclonus status epilepticus within the first 24 hours, absence of pupillary responses within days 1 to 3 after CPR, absence of corneal reflexes within days 1 to 3 after CA, and absent or extensor motor responses after 3 days [37,38]. Thus, the prognosis is invariably poor in comatose patients with absent pupillary or corneal reflexes, or absent or extensor motor responses 3 days after CA. On the opposite, single seizures and sporadic focal myoclonus do not accurately predict poor outcome. In the same way, although permanent status epilepticus is associated with a mortality approaching 100%, exceptions have been reported.

## Investigations that may enhance prognostication

In addition to physical examination, various methods have been evaluated for assessing the neurological prognosis of comatose CA victims. Electrophysiological tests in coma consist of EEG and evoked/event-related potential studies. Somatosensory evoked potentials (SSEPs) are the best diagnostic method for predicting outcome in comatose patients. Bilateral absence of the N20 component of the SSEPs with median nerve stimulation recorded on days 1 to 3 or later after CA accurately predicts a poor outcome [38]. Conversely, the presence of the N20 response is not helpful to predict outcome as many patients who fail to recover can have preserved N20 responses.

Routine EEG examinations are not supported by the results of the published reviews but should be used if ongoing seizure activity is suspected. Generalized suppression pattern, burst-suppression pattern with generalized epileptiform activity, or generalized periodic complexes on a flat background are strongly but not invariably associated with poor outcome [38].

Anoxo-ischemic cerebral insult is associated with the blood release of various biomarkers and the peak plasmatic level is thought to be correlated with the amount of neuronal death. A large serum peak of neuron-specific enolase and/or S-100 protein is highly specific but only moderately sensitive for predicting a poor neurological outcome [38]. The search for a simple, reliable, and readily available biological test remains an exciting challenge [39], but clinical decisions with potentially irreversible consequences should never rely on a single marker.

Regarding routine neuroimaging techniques, CT-scan imaging adds no information unless stroke, bleeding, or trauma is suspected. Preliminary reports suggest that magnetic resonance imaging could be used to determine the prognosis of patients with diffuse cerebral anoxia [40] but warrants further investigations.

## End-of-life process after cardiac arrest

In a small number of distressing cases, patients regain spontaneous circulation but remain in a persistent vegetative state. It can be stated that continued existence of this state is not in the patient's best interest even if the only other alternative remains death. When this approach is shared both by care providers and relatives, end of life care must be considered: this consists in withholding/withdrawing all invasive and supportive care. Considering that most of these neurologically impaired patients are mechanically ventilated, the decision to withdraw therapies will nearly always include a statement about the pursuit of this assistance. When all conditions are joined, it is suitable to plan an extubation and the definitive withdrawal of respiratory assistance. This should be performed in conjunction with all comfort care that can be provided in such a situation (pain treatment, sedation). Decision to withdraw life-support treatment and the modalities of this withdrawal must be the conclusion of a collegial process between team and proxies, and all must be made to avoid the family's feeling of responsibility. Such decisions must obviously meet all the legal steps that exist in the country.

## Mild and long-term outcome of survivors

### Recurrence prevention

Studies that focus on patient outcome beyond the first month after hospital discharge are limited. Historical data are hardly comparable to the populations who have benefited from recent progress in term of access to early defibrillation and ICU management. The long-term mortality is generally considered satisfactory, with a 5-year survival of approximately 80% for patients leaving ICU after CA due to ventricular arrhythmia [41]. This is slightly lower than subjects of similar age but is similar to patients of identical age and matched with comorbidities.

**Figure 3:**
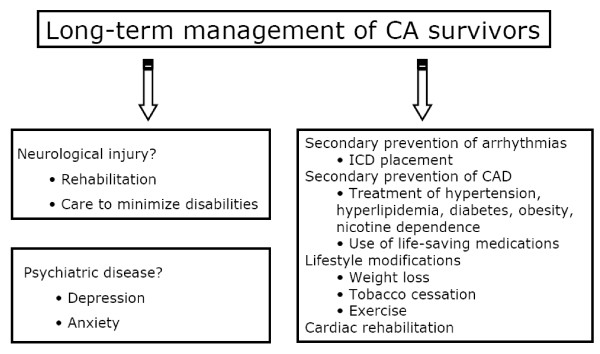
**Long-term pitfalls in cardiac arrest survivors**.

The cardiac outcome, and primarily the risk for recurrence of sudden death, is obviously different depending on the etiology of initial CA. Patients who experienced ventricular arrhythmia have been widely studied because of the availability of implantable cardioverter defibrillator (ICD). This last one significantly reduces the risk of sudden death compared with antiarrhythmic drugs [42,43], demonstrating clearly the benefit of ICD in patients resuscitated from a ventricular arrhythmia. The majority of cardiovascular mortality (including sudden death) is observed within 12 to 24 months after the initial arrhythmia [44]. During this period, systolic function is the best predictor of early recurrence. CA survivors should therefore benefit from a comprehensive etiological investigation. The depth and the means for assessing the patient's cardiovascular status and long-term risk prognosis are obviously largely dependent on the magnitude of neurological recovery. Other therapeutic modalities are common with patients experiencing cardiovascular disease, especially in patients with ischemic disease, who constitute the majority of the survivors (Figure 3).

### Sequels and quality of life

Beyond recurrence, quality of life and the possibility of finding a social activity in patients sometimes young and active often are poorly taken into consideration. Of the 681 patients studied after discharge from hospital for a period of 6 months, 69% of them were considered to have a good neurological recovery at discharge [45]. For 70% of patients followed, the neurological status remained stable, or even improved, in 12% of cases; only 1% of the patients exhibited a decrease in neurological performance. In this cohort, the 6-month mortality of 17% was mainly due to cardiovascular causes. To go further, a fine evaluation of the quality of life was performed 3 years after resuscitation from ventricular fibrillation, using the standardized questionnaire SF-36 [41], which explores both physical and mental aspects. Results were superposable on the one of a matched population, except that survivors displayed a lower sense of vitality. Hypoxic insult may nevertheless result in a number of disorders considered as minor but having a strong impact on quality of life, such as memory problems. They may be even more frequent in younger patients than in older subjects. Overall, health status of these patients is therefore considered as satisfactory [46,47].

In recent years, interest in posttraumatic stress disorder (PTSD) has steadily increased in patients experiencing ICU admission [48]. CA survivors represent an important potential source for development of PTSD. A study compared the psychological status of patients who experienced intrahospital cardiac arrest or a myocardial infarction 9 months after the event and revealed that, among survivors of cardiac arrest, 30% suffered from anxiety, 15% depression, and 19% PTSD, whereas the proportions observed were respectively 7%, 0%, and 7% after acute myocardial infarction [49]. This study highlights the frailty of survivors of cardiac arrest and the significant risk of emotional disorders [50]. Another study showed that PTSD occurred in one third of survivors of CA 45 months after the event [51]. Interestingly, this study displayed that the only risk factor for PTSD was age: the younger the patient, the more at risk he is to suffer from PTSD. Conversely, according to the study, pre- and intrahospital management of cardiac arrest did not affect the onset of PTSD.

## Conclusions

Major progress has occurred during the past 10 years in the initial management of successfully resuscitated CA: the development of a "chain of survival," the availability of automated external defibrillators in paramedical teams and public places, and the innovations in the resuscitation process gives hope for a further improvement in early survival. This will be accompanied by a steady increase in patients exposed to postcardiac-arrest syndrome. A better understanding of this syndrome undoubtedly transforms the last link of the survival chain: from an attitude of observation, the hospital management now becomes active and might condition in part the patient's progress in the short and long term. The primary objective of such care is to obtain survival with no or little neurological sequels. By influencing the vital and functional prognosis of patients, cerebral protection is now an essential part of the management of postcardiac-arrest patients. Currently, it relies mainly on therapeutic hypothermia. In the future, new pharmacological and nonpharmacological treatments should contribute to further improve the prognosis of these patients.

## Competing interests

The authors declare that they have no competing interests.

## Authors' contributions

NM and AC drafted the manuscript. All authors read and approved the final manuscript.
